# No association of polymorphisms in the serotonin transporter gene with thermal pain sensation in healthy individuals

**DOI:** 10.1186/1744-8069-10-76

**Published:** 2014-12-04

**Authors:** Ellen Lund Schaldemose, Emilia Horjales-Araujo, Ditte Demontis, Anders D Børglum, Peter Svensson, Nanna Brix Finnerup

**Affiliations:** Danish Pain Research Center, Aarhus University Hospital, Norrebrogade 44, Building 1A, DK-8000 Aarhus C, Denmark; Department of Biomedicine and Center for Integrative Sequencing (iSEQ), Aarhus University, DK-8000 Aarhus C, Denmark; Section of Clinical Oral Physiology, Department of Dentistry, Aarhus University, Aarhus, Denmark; Scandinavian Center for Orofacial Neurosciences (SCON), DK-8000 Aarhus C, Denmark

**Keywords:** Serotonin transporter, Capsaicin, Cold, Warm, Pain, Thermal grill

## Abstract

**Background:**

Recent studies have suggested an association between genotypes affecting the expression of the serotonin transporter and thermal pain perception and the thermal grill. The aim of this study was to investigate differences in thermal and mechanical pain perception and the thermal grill in two groups of healthy volunteers according to their genotype, associated with either high (n = 40) or low (n = 40) expression of the serotonin transporter and according to gender. Cold and warm detection and pain thresholds, pressure pain threshold and cold, warm and pain sensations to single or alternating stimuli with cold (20°C) and warm (40°C) temperatures (known as the thermal grill) were determined. In addition, intensity of ongoing pain and area and intensity of pinprick hyperalgesia in the secondary hyperalgesic area following topical application of capsaicin and vehicle control (ethanol) were determined.

**Results:**

No significant differences in detection and pain thresholds for cold and warm temperatures, presence of paradoxical heat sensation, pressure pain threshold and pain responses to suprathreshold thermal stimuli were observed. There was also no difference in capsaicin-evoked ongoing pain and secondary hyperalgesia between the two genotype groups (p >0.4), also when subdivided by gender (p >0.17). In addition, there were no significant differences in the perception of the thermal grill between the two genotypes (p >0.5), also when subdivided by gender.

**Conclusions:**

Genotypes associated with high or low expression of the serotonin transporter were not associated with thermal pain thresholds, pressure pain threshold, pain after capsaicin application or responses to the thermal grill.

The present results do not support that the investigated genotypes play a major role in thermal pain perception among healthy individuals.

## Background

Pain is a subjective experience that is influenced by a combination of factors such as gender, genes, emotions, sensations and culture [1, 2], and its expression therefore varies widely between individuals [3].

One of the neurotransmitters that has been associated with pain is serotonin (5-hydroxytryptamine, 5-HT) [4]. An important regulator of 5-HT signaling is the serotonin transporter (5-HTT), which removes 5-HT from the synaptic cleft through reuptake and terminates the extracellular signaling [5]. Recent studies have demonstrated associations between polymorphisms in the serotonin transporter gene that affect the expression of the 5-HTT and pain modulation [6–8], response to the opioid remifentanil [9] and thermal pain sensitivity [10]. In humans, the 5-HTT is coded by a single gene (*SLC6A4*) located on chromosome 17. The promoter region of the gene encompasses a polymorphic region with a 44-bp insertion/deletion of a C/G-rich variable number of tandem repeat sequence, referred to as the 5-HTT linked polymorphic region (5-HTTLPR). This insertion/deletion generates a long (L) or a short (S) allele. The S-allele is related to lower transcriptional efficacy of the gene and thereby decreased expression of the 5-HTT. Furthermore, a single nucleotide polymorphism (SNP), labeled rs25531, located in the promoter region of *SLC6A4* is known to alter the degree of expression [11, 12]. This SNP consists of an A to G substitution. The G-allele is most frequently coupled to the L-allele of the 5-HTTLPR and is suggested to reduce the transcriptional efficacy to levels analogous to the S-allele. The 5-HTTLPR together with rs25531 is termed the “triallelic” 5-HTTLPR and is functionally divided in individuals having genotypes known to be associated with high (L_A_/L_A_), intermediate (L_G_ /L_A_, L_A_ /S_A_) or low expression (S_A_/S_A_, L_G_/S_A_, L_G_/L_G_) of the 5-HTT.

In a previous study, low 5-HTT expression was associated specifically with decreased sensitivity to heat and cold pain, i.e. increased thermal pain thresholds, in 44 healthy volunteers [10]. However, a subsequent study from the same group in 45 healthy subjects failed to find a difference in heat pain thresholds between those with low and those with high 5-HTT expression [6]. The low 5-HTT expressing group had lower pain ratings to heat stimuli of 46°C, but not to 47°C and 48°C, and the difference was not statistically significant [6]. While Potvin and colleagues [13] failed to find a relationship between the 5-HTTLPR and thermal pain thresholds in 60 healthy participants and 58 fibromyalgia patients, Hooten and colleagues [14] reported higher heat pain thresholds in 277 chronic pain patients with the intermediate expression genotype compared with a high expression group.

Since the relationship between thermal sensitivity and 5-HTT expression remains unclear, we aimed to investigate the association between polymorphisms in the triallelic 5-HTTLPR and different sensory characteristics in a larger group of 80 healthy non-depressed individuals. Thermal thresholds can be assessed using quantitative sensory testing (QST). In addition, capsaicin (the substance responsible for the burning sensation in “hot” chili peppers), can be used as a model of acute thermal pain. Capsaicin activates the transient receptor vanilloid 1 (TRPV1) receptor expressed in subtypes of C-fiber nociceptors and Aδ fibers [15] producing both primary and secondary hyperalgesia as well as allodynia to heat and mechanical stimuli [16, 17]. The so-called ”thermal grill (TG) illusion”, first demonstrated by Thorsten Thunberg in 1898, is a thermal phenomenon of heat or itching cold and eventually pain elicited by touching the skin with juxtapositioned innocuous warm and cold bars [18, 19]. Studies in healthy volunteers have shown large inter-individual differences, with almost one-third reported to be low or poor responders [20, 21]. This difference is suggested to be due to common genetic variations among the volunteers, e.g. in 5-HTTLPR [10]. Lindstedt and colleagues found a gender-by-genotype interaction with significantly lower unpleasantness ratings to the TG in females in a low 5-HTT-expression group compared with females in a high expression group [10].

The overall aim of the present study was to investigate differences in selected sensory parameters in participants with genotypes associated with high or low expression of the 5-HTT. Based on former studies we chose to asses thermal detection and pain thresholds, pressure pain threshold, pain response to topical application of capsaicin and ethanol (control) and sensory responses to the TG in 80 healthy, non-depressed individuals [10, 14]. In addition the aim was to examine possible gender specific differences. We hypothesized that participants with low-expressing 5-HTT genotypes have higher heat and cold pain thresholds and decreased pain responses to thermal stimuli, capsaicin and the TG.

In summary, detection and pain thresholds for cold and warm temperatures, pressure pain threshold, pain ratings of topical application of capsaicin and the sensory dimensions of the TG were determined.

## Results

**Figure 1:**
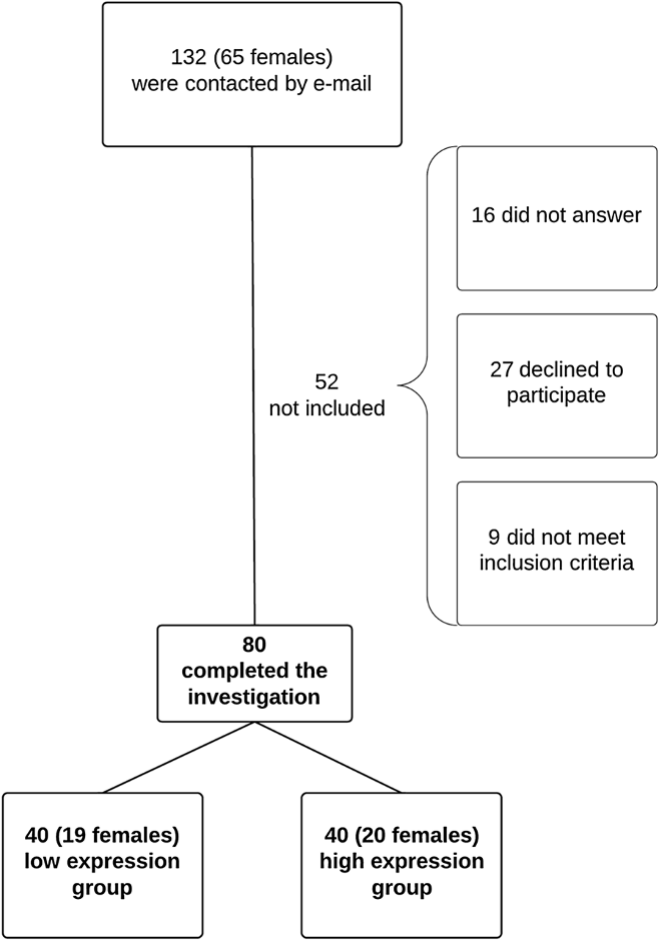
**Flowchart of recruitment.**

Forty participants were included in each group (19 women in the low expression group and 20 women in the high expression group) (Figure 1). No participants dropped out. There were no significant demographic or clinical differences between the two groups (p >0.67, *t*-test) (Table 1).

**Table 1:** **Demographic and clinical characteristics of participants**

	Low 5-HTT expression	High 5-HTT expression	P-value
**Demographic characteristics**			
N (men:women)	40 (21:19)	40 (20:20)	0.82^a^
Age, mean ± SD (minimum-maximum), year	25 ± 2.3 (19–31)	25 ± 2.4 (19–32)	0.67^a^
**Clinical data**			
**Major depression inventory (MDI)**			
MDI score mean ± SD (minimum-maximum)	5.5 ± 3.0 (0–13)	5.3 ± 3.1 (0–13)	0.74^a^
Moderate or severe depression, N	0	0	
**General anxiety disorder (GAD)**			
GAD score mean ± SD (minimum-maximum)	3.1 ± 2.9 (0–13)	3.1 ± 2.2 (0–7)	0.93^a^
Doubtful anxiety disorder	2	0	

^a^ *t*-test.

MDI scores: 0–21 = “no depression”, 26–30 = “moderate depression”, 31 or more = “severe depression”.

GAD scores: 0–7 = ” no anxiety disorder”, 8–14 = ” doubtful anxiety disorder”, 15–19 = ” mild anxiety disorder”, 20–29 = ” moderate anxiety disorder”, 30–50 = ” severe anxiety disorder”.

### Quantitative sensory testing

**Figure 2:**
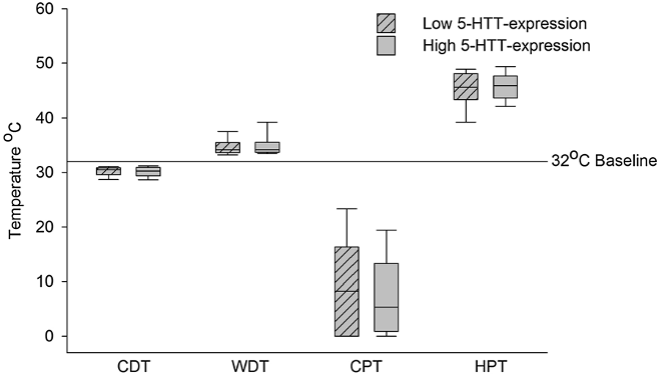
**Thermal detection and pain thresholds, divided by triallelic 5-HTTLPR.** CDT: cold detection threshold; WDT: warm detection threshold; CPT: cold pain threshold, HPT: heat pain threshold. Boxes: interquartile range, whiskers: 10% and 90% percentiles. No significant differences between groups (p >0.4 Mann–Whitney U-test).

No significant differences in detection and pain thresholds for cold and warm temperatures were observed between the two genotype groups (p >0.41, Mann Whitney U-test) (Figure 2, Table 2). Additionally, no differences were found in baseline skin temperature (p =0.47, Mann–Whitney U-test), pressure pain threshold (p =0.41, Mann–Whitney U-test) and number of paradoxical heat sensations (p =0.64, Pearson’s χ^2^ test). We found no significant differences in the above-mentioned parameters when the groups were divided by gender (p ≥0.27 (males); p ≥0.34 (females)).

**Table 2:** **Baseline QST, sensory intensities following capsaicin/ethanol, and area of hyperalgesia and responses to pinprick stimuli in the secondary hyperalgesic area following capsaicin/ethanol**

	Low 5-HTT expression	High 5-HTT expression	p-value
**Skin temperature, °C**	32.10 (30.95-32.3)	31.4 (31–32.2)	0.47^a^
**Quantitative sensory testing**			
CDT, °C	30.49 (29.65-30.87)	30.25 (29.38-30.92)	0.89^a^
WDT, °C	34.19 (33.65-35.42)	34.2 (33.7-35.5)	0.83^a^
CPT, °C	8.3 (0.0-16.2)	5.3 (0.9-12.6)	0.48^a^
HPT, °C	45.59 (43.44-48.05)	45.85 (43.7-47.55)	0.96^a^
PPT, kPa	506.5 (423.5-678.5)	458.0 (425–597)	0.41^a^
PHS, N/N	2/40	3/40	0.51^b^
**Sensory intensities following capsaicin/ethanol, VAS area under the curve (AUC)**			
**Capsaicin**			
Pain, cm^2^	262.8 (29–508)	266 (79.8-467)	0.98^a^
Unpleasantness, cm^2^	437.8 (251.3-825.8)	520 (276–698.5)	0.89^a^
**Ethanol (control)**			
Pain, cm^2^	0 (0–0)	0 (0–0)	0.34^a^
Unpleasantness, cm^2^	0 (0–0)	0 (0–0)	0.45^a^
**Area of hyperalgesia**			
**Capsaicin, cm** ^**2**^	3 (0–10)	4 (0–11)	0.93^a^
**Ethanol (control), cm** ^**2**^	0 (0–0)	0 (0–2)	0.37^a^
**Pinprick**			
**Capsaicin**			
Pain, VAS	0 (0–8)	0 (0–1.5)	0.24^a^
Unpleasantness, VAS	9 (0–22.5)	4 (0–17.5)	0.20^a^
**Ethanol (control)**			
Pain, VAS	0 (0–0)	0 (0–0)	0.47^a^
Unpleasantness, VAS	0 (0–5.5)	0 (0–1)	0.17^a^

^a^Mann–Whitney U-test, ^b^Fisher’s exact test.

Data are presented as median (interquartile range). *CDT* cold detection threshold, *WDT* warm detection threshold, *CPT* cold pain threshold, *HPT* heat pain threshold, *PPT* pressure pain threshold, *PHS* paradoxical heat sensations. Numbers of PHS are presented as responders/participants.

### Suprathreshold

Temperatures at 15°C, 25°C and 35°C were not rated as painful and were therefore not analyzed. We found no significant differences in pain ratings at 5°C, 42°C and 45°C (p >0.14, Mann–Whitney U-test). Additionally there were no differences when the participants were divided by gender and genotype. χ^2^ tests were conducted for the temperature descriptions at the specific temperatures: 5°C (freezing cold and cold), 15°C (cold and neutral), 25°C (cold and neutral), 35°C (neutral and hot), 42°C (hot and burning hot) and 45°C (hot and burning hot). We found no significant differences between the two groups except a more neutral ranking (instead of warm) at 35°C in the low 5-HTT expression group (p =0.02, χ^2^ test). There were no significant differences for the pain ratings when dividing by gender and genotype (p ≥0.29 (males); p ≥0.31 (females)).

### Thermal grill

**Figure 3:**
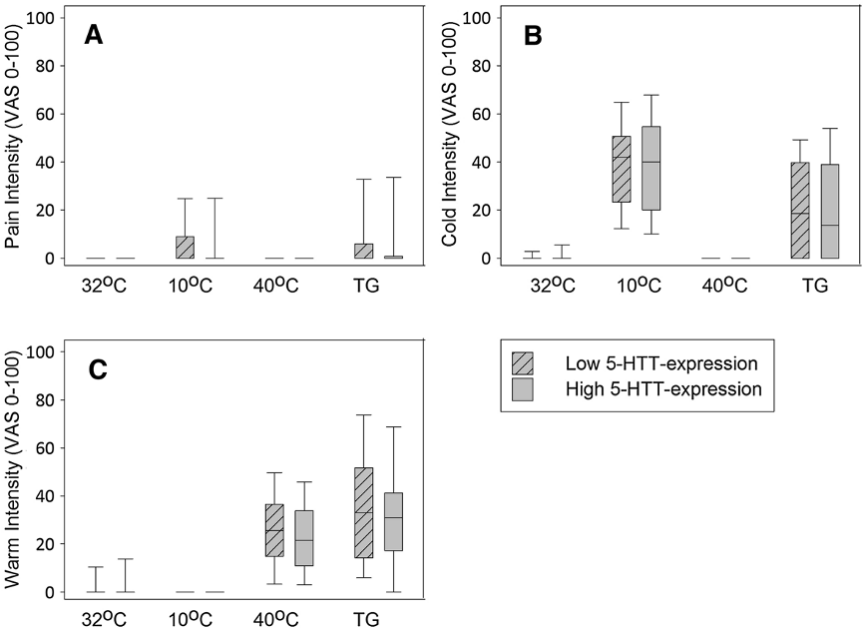
**Pain, cold and warm intensities during the Thermal Grill testing, divided by triallelic 5-HTTLPR.** Intensity of pain **(A)**, cold **(B)** and warm **(C)** (VAS 0–100) to stimulation with 32°C, 10°C, 40°C and TG (10/40°C). Boxes: interquartile range, whiskers: 10% and 90% percentiles. No significant differences between groups (p >0.5, Mann–Whitney U-test).

Figure 3, A-C shows pain, cold and warm intensities (on a 0–100 Visual Analog Scale (VAS)) at 32°C, 10°C, 40°C and TG (10°C/40°C) grouped by genotype. At any given temperature there were no significant differences in the VAS ratings of pain or temperature between the two genotype groups (p ≥0.15, Mann Whitney U-test). When divided by gender we still found no differences (p ≥0.14 (males); p ≥0.14 (females)). No significant differences were found in the temperature descriptions between the two groups overall or when dividing by gender and genotype (p ≥0.22 (both); p ≥0.15 (males); p ≥0.14 (females)).

To elucidate any difference in the TG response between the genotype groups, we compared TG responders with TG non-responders. We defined participants to be responders in two ways: 1) participants who rated the TG condition but not 40°C stimuli as burning hot and/or 2) participants having a higher VAS pain rating in the TG condition compared with stimuli of 10°C or 40°C (Table 3). We also found no significant differences between the groups when dividing by gender and genotype (p >0.16, Fisher’s exact).

**Table 3:** **Thermal grill responders**

TG responders	Low 5-HTT expression	High 5-HTT expression	P-value
**Thermal grill burning hot**			
TG responder	10 (7 females)	6 (3 females)	0.40^a^
TG non-responder	30 (12 females)	34 (17 females)	
**Thermal grill painful**			
TG responder	10 (5 females)	5 (2 females)	0.52^a^
TG non-responder	30 (14 females)	35 (18 females)	

^a^Fisher’s exact.

Number of TG responders defined either by 1) participants ranking TG condition (and not 40°C) as burning hot and 2) participants having higher pain ratings during the TG condition than during 10 or 40°C.

### Capsaicin and ethanol

Application of capsaicin to the skin evoked moderate levels of pain and unpleasantness in all participants (VAS mean pain to 28-min capsaicin application: 23 (5.5-43); VAS mean unpleasantness to 28-min capsaicin application: 31.5 (17–45)). The control condition (ethanol) elicited no pain and very low levels of unpleasantness (VAS mean pain to 28-min ethanol application: 0 (0–0); VAS mean unpleasantness to 28-min ethanol application: 0 (0–0)). We found no significant differences in VAS area under the curve neither for pain nor for unpleasantness between the two genotype groups (Table 2), also when subdivided by gender and genotype (p ≥0.34 (both); p ≥0.21 (males); p ≥0.17 (females)).

### Pinprick, brush stimuli and area of hyperalgesia

There was no brush allodynia following the capsaicin application. We found a significantly larger area of pinprick hyperalgesia after capsaicin application compared with ethanol application (p <0.001, Wilcoxon signed-rank test), but no difference between the genotype groups (p ≥0.37, Mann–Whitney U-test) (Table 2). Additionally we found no difference in intensity of pinprick hyperalgesia (Table 2).

## Discussion

Our main findings were that thresholds to thermal pain sensitivity and pain intensity and pressure pain threshold were not associated with the triallelic 5-HTTLPR. In addition, we found no differences in spontaneous pain and secondary hyperalgesia and allodynia following capsaicin application and no difference in the TG assessments between participants with low and high 5-HTT expression, even when dividing into females and males. Interestingly, these results appear to be in contradiction to a previous study indicating that a cautious position should be taken when exploring associations between specific genotypes and pain [10], and replication in additional studies should be undertaken before any conclusion can be made. Lindstedt and colleagues reported a significantly reduced sensitivity to heat and cold pain in a low 5-HTT expression group (n = 21) compared with a high expression group (n = 23). Additionally, they found a gender-by-genotype effect with lower unpleasantness ratings to the TG in low 5-HTT expressing females compared with high 5-HTT expressing females [10]. During the TG, we did not assess unpleasantness, but we found no difference in thermal and pain ratings to the TG between the groups, also not in females. The initial burning sensation of capsaicin is comparable to heat-induced pain [22]. We found no difference in pain ratings for capsaicin between the genotype groups. Additionally, we observed no difference in the area and intensity of secondary pinprick hyperalgesia, suggesting no difference in the extent of central sensitization. Our study population was slightly younger than the healthy controls included in the mentioned study, and it is possible that the discrepancy might be explained by age-related changes in thermal pain perception. However, we find this unlikely, since the difference in age was small, and we found no tendency in our material that went in the same direction as the results in the Lindstedt study. Our results are thus in accordance with other studies that have failed to find a reduced sensitivity to heat and cold pain in the low 5-HTT expression group [6, 9, 14].

In previous genetic studies associating polymorphisms in the serotonin transporter gene with heat pain in healthy individuals, the number of involved participants in each genotype group have been around 20 (12–25 participants) [9, 10, 13]. To the best of our knowledge, this is the first report testing the hypothesis in a considerably larger sample. Even though our sample size is higher than in previous studies, it is still a rather low number when considering that we are working with a polygenic trait in which each contributing genetic variant may only contribute little to the resulting phenotype. The lack of association of the triallelic 5-HTTLPR with the pain-related tests in this study does therefore not exclude that genotypes affecting the expression of the serotonin transporter are associated with the investigated traits. The lack of significant association could be due to a low effect size of the investigated genotypes on the traits, which might only reveal a significant association in a larger sample.

To our knowledge this is the first study investigating the association of the pressure pain thresholds and polymorphisms in the serotonin transporter. We did not find an association indicating that differences in mechanical pressure pain thresholds is associated with the triallelic 5-HTTLPR. This is possible because variations in many genetic polymorphisms affects the pressure pain threshold or simply because difference in genotypes affecting the 5-HTT expression has no influence on pressure pain threshold.

We did not control for the women’s menstrual phases which possible could lead to higher variable data for the female participants. It is suggested that women’s pain sensitivity fluctuate with the menstrual phases, with a higher pain threshold in the mid-follicular phase compared with the luteal phase. Studies testing different pain characteristics found no significant intra-individual difference in thermal pain thresholds between the luteal, midfollicular and ovulatory phase ((n = 11), (n = 32)) [23, 24]. Since the number of women in the two groups is the same and it is likely that the distribution of menstrual phases is balanced [10] and the fluctuation in pain sensitivity is relatively small [25] the possible effects of the menstrual phases on the difference in pain thresholds are small.

Pain sensation should be considered a polygenic trait, and many genes are involved in the regulation of nociceptive activity. Differences in regulation could therefore be affected by genetic variations in coding and regulatory regions of many genes as well as their mutual interaction. Besides the genetic variation that affects the expression of the serotonin transporter, genetic variability in different subtypes of serotonin receptors have also been suggested to be involved in pain regulation. For instance, low expression of the 5-HT_1A_-receptor and different SNPs in the receptor gene (*HTR1A*) have been associated with altered thermal pain thresholds in 49 healthy volunteers [26]. In 34 patients with polyneuropathy, it has been suggested that variations in the gene coding for the 5-HT_2c_-receptor are associated with differences in the pain-relieving effect of escitalopram [27]. Because of the interaction of the serotonin transporter and serotonin receptors, it is most likely that thermal pain sensation is affected by many genetic polymorphisms in the pain-regulating pathways. Furthermore, the study by Horjales-Araujo et al. [7] has demonstrated that environmental factors/external emotional stimuli can affect the perception of pain, which also states that gene-environment interactions may influence pain sensation thresholds.

## Conclusion

We found no association between thermal pain thresholds, pressure pain thresholds, pain after capsaicin application, response to the TG and polymorphisms in the triallelic 5-HTTLPR. While the expression of the serotonin transporter may still be of importance in shaping the clinical manifestations of thermal pain, the present results also indicate that the gene-environment interaction of pain may be more complex than a single pain candidate gene.

## Methods

### Participants

A total of 80 healthy participants were selected on the basis of their polymorphism in the serotonin transporter gene from a genetic database of 380 individuals. The participants had been genotyped for the triallelic 5-HTTLPR in a previous study and had given consent to be contacted for future studies [7]. Forty participants with low expression genotypes of the serotonin transporter (S_A_/S_A_, L_G_/S_A_, L_G_/L_G_) and 40 participants with high expression (L_A_/L_A_) of the gene, were included in the study (Figure 1). Other inclusion criteria were age between 18 and 39 years and Scandinavian descent [7]. None of the included participants had any known neurological, psychological or cardiovascular disorder or chronic pain condition, used regular medicine (except contraceptives), were smokers or were pregnant. We did not obtain information on the women’s menstrual cycle. The study was approved by the local ethical committee (1-10-72-165-13), the Danish Data Protection Agency (2007-58-0010) and conducted according to the Declaration of Helsinki II. The participants gave their written informed consent and received 500 DKK as compensation.

### Study design

The participants completed the General Anxiety Disorder (GAD) questionnaire and the Major Depression Inventory (MDI) [28]. Quantitative sensory testing, the sensory dimensions of the thermal grill illusion and pain and unpleasantness caused by application of capsaicin were examined. All tests were performed randomly on the participants’ right or left ventral forearm and by the same investigator. Both the investigator and the participants were blinded for the genotype of the participants.

### DNA analysis

DNA analysis was done as part of previous studies [7, 29]. In short, participants’ DNA was extracted from saliva collected using an OC-100 kit (DNA Genotek Inc, Ontario, Canada). To determine the triallelic 5-HTTLPR genotype, PCR reactions were carried out in a total volume of 25 μl using the GoTaq® Hot Start Polymerase (Promega, Wisconsin, USA) and 80 ng of genomic template. The forward primer sequence was 5′-CTCTGAATGCCAGCACCTAACCC-3′ and the reverse 5′-GATTCTGGTGCCACCTAGACGC-3′. Samples were amplified (Gene Amp, PCR System 9700, Applied Biosystems, California, USA) by 2-step PCR consisting of an activation step of 2 min at 94°C, followed by 35 cycles of 30 s denaturation at 93°C, and an annealing and elongation step for 1 min at 62°C, followed by a final elongation step of 10 min at 72°C. Respectively, the L-allele and S-allele of the 5-HTTLPR yield a product of 529 bp and 486 bp. Fragments were visualized with UV after 45-min separation at 80 V on a 2.5% agarose gel. To determine the rs25531 polymorphism, 10 μl of the PCR product was digested for 2 h at 37°C with 1 μl MSP1 (New England Biolabs, Ipswich, MA, USA) and 1 μl buffer per sample. The enzyme cuts at a 5′-C/CGC-3′sequence, resulting in fragments of different length which determined the triallelic genotype. The digested fragments were visualized by UV light after 2-h separation at 100 V on a 4% agarose gel.

### Quantitative sensory testing (QST)

Thermal testing and measurement of pressure pain were performed using the QST protocol from the German Research Network on Neuropathic Pain (DFNS) [30]. The DFNS QST protocol uses standardized equipment and verbal instructions and our center is certified according to the DFNS standards. Prior to the QST, skin temperature was measured using a digital infrared temperature scanner (Omega OS90 series).

Thermal tests were conducted using the thermal sensory analyzer (TSA, Medoc, Israel). The participants were introduced to the method and underwent a pre-test. The investigator recited the same information to every participant and they were told to look away from the test side. First, the detection thresholds for alternating cold and warm temperatures were measured. To register any possible paradoxical heat sensation, the participants were asked to report the sensation of the temperature (either cold or warm). Next, cold and warm detection thresholds (CDT and WDT) and finally the pain thresholds for cold (CPT) and heat (HPT) were measured. The thresholds were reached by continuously increasing/decreasing the temperature by 1°C/s and terminated when the participants pressed a button. Before each test the thermode returned to a baseline temperature of 32°C. The contact area was 3x3 cm and the cut-off temperatures were 0°C and 50°C. The mean threshold value of three consecutive measurements was calculated. Additionally we assessed the stimulus response to thermal stimuli temperatures at 5°C, 15°C, 25°C, 35°C, 42°C and 45°C in randomized order. Each stimulus was applied for approximately 5 s, after which the participants rated potential pain on the VAS (0–100) and characterized the sensation as either burning hot, warm, neutral, cold or freezing cold. There was a 10-s pause between each stimulus.

A pressure gauge device (Wagner instruments, Greenwich, USA) was used to measure pressure pain thresholds over the muscles of the thenar eminence. The pressure was gradually increased by approximate 0.5 kg/s (≈50 kPa/s) and terminated when the participants reported a painful sensation. Mean of three consecutive pressures was calculated. All QST measurements were assessed with the participants in a sitting position.

### Thermal grill

A thermal grill, developed by Somedic, Sweden, was used. The grill consists of 8 rectangular thin silver plates (80 mm × 10 mm × 1 mm) housed in a polyvinylchloride unit. The temperature of odd and even numbered silver plates were controlled separately using circulating water from two baths, one used for cooling and one used for heating. A switch allowed the circulation to be set so that odd and even numbered bars were either held at the same temperature (cold or warm) or alternated between cold and warm temperatures (i.e. thermal grill condition). The system was calibrated to achieve approximately 40°C and/or 10°C at the silver plates. This relatively large temperature interval between the cold and the warm bars was chosen to induce a moderately intense thermal grill condition [20]. A randomization list was used to achieve a counterbalanced order between the three conditions, i.e. cold only, warm only and cold and warm (= TG). The silver plates were set to the correct condition before skin contact. Participants used the same arm they used for the QST. They were asked to place their arm on the thermal grill perpendicular to the long axis of the silver bars a total of six times, 20 s each time (three tests and three neutral conditions). Immediately after each stimulus, the participants characterized the temperature as either burning hot, hot, neutral, cold or freezing cold, and they were asked to rate potential pain and intensity of cold and warm stimuli on a separate 0–100 VAS. To neutralize the skin area, a stimulus of 32°C for 20 s was used before and between each stimulus. The participants did not get any information about the mechanism, only that the temperatures could be painful but not injurious. Because of the cooling equipment (especially the TG), a background noise was present throughout the investigation. The TG was performed with the participants in standing position.

### Capsaicin

A 0.5 ml solution of capsaicin (0.9 mg/ml, 70% ethanol) or vehicle control (ethanol, 70%) was applied to the skin surface for 28 min. The solution was dispersed equally at two plasters (Tegaderm + Pad, 3M) with a total stimulus area of 8.4 × 2.6 cm. One arm was treated with capsaicin and the other with ethanol as vehicle; the order and the side were assigned according to a computer-generated randomization list and were not revealed to the participants. An interval of 9 min between the two sides was held, always starting with the right arm. The capsaicin dose was chosen on the basis of preliminary tests and previous studies [31, 32]. The aim was to achieve a concentration that induced pain, but still allowed the participants to distinguish between the different temperatures, especially with respect to the TG assessments.

During the first 20 min of the capsaicin or ethanol application, the skin was warmed with a heating lamp to a skin temperature of 36 ± 0.5°C to maintain a continuous capsaicin-induced pain [16]. The skin temperature was monitored with an interval of 5 min during the assessment.

Participants scored the intensity of pain and unpleasantness every fifth minute during the first 20 min and after 28 min. The perceived intensities of pain and unpleasantness were calculated as the area under the curve. The heating lamp was removed after 20 min and the area of mechanical hyperalgesia was determined. Subsequently, the participants were asked to rate the intensity of pain and unpleasantness on the VAS to pinprick and brush stimuli at the area they reported as most unpleasant/painful. The plasters were removed after 28 min.

### Area of hyperalgesia to pinprick stimuli

Stimuli were induced by a monofilament (Semmes-Weinstein, Stoelting, IL) with an estimated force of 745 mN [33]. The filament was applied at least 3 cm from the periphery of the vehicle area, and the stimulus was repeated closer and closer to the area along a line perpendicular to the plasters, with a distance of 1 cm between each dot. The testing area was mapped with marking dots on the skin with a felt tip pen before testing. A total of 14 repetitions were made, with a distance of 2 cm between each, and terminated when the participants reported an increase in sensitivity [34]. The participants were told to look away from the testing area and were instructed to report any change in sensation after each repetition, e.g. increase in intensity, pain and pricking.

The area (*A*) of hyperalgesia was calculated by adding the areas of the 14 single repetitions, i.e. *A* = ∑*h*_*i*_ x 2 where *h*_*i*_ is the distance from a spot reported as more intense (*h*_*1*_ + *h*_*2*_ + *h*_*3*_ + … + *h*_*14*_ ) and “*2*” the distance between each repetition (in cm) [35].

### Pinprick hyperalgesia and brush allodynia

We used the same monofilament for the pinprick stimuli as for the measurement of area of hyperalgesia. A standardized brush (Somedic, Sweden, estimated force of 200-400mN) were used for the brush stimuli. The stimuli were applied with a single stroke of approximately 2 cm in length over the skin. Both pinprick and brush stimuli were applied at the area the participants reported as most unpleasant/painful and they rated intensity of pain and unpleasantness (VAS). Measurements were performed at both arms, and the participants were told to look away from the application site during the assessment.

### Statistics

Stata for Windows (version 11.2) was used for data analysis. The data were visually inspected as histograms with corresponding normal curve to test for departure of normality. Since the majority of the measurements were non-parametric, data are reported as medians and interquartile range. The demographic and clinical data are presented as mean ± standard deviation. The Mann–Whitney *U-*test was used to compare the two groups. Pearson’s χ^2^ and Fisher’s exact were used on categorical variables between the groups. P-values <0.05 were considered significant.

Power calculations were done prior to recruitment. Based on a previous study showing a difference of 2.2°C in heat pain threshold we aimed to find a difference of 1.8°C in thermal thresholds [10]. With 39 individuals in each group we would achieve a statistical power >80% with α = 0.05.

## Abbreviations

5-HT: 5-hydroxytryptamine/serotonin
5-HT_1A_: Serotonin receptor 1A
HTR1A: Serotonin receptor 1A gene
5-HT_2c_: Serotonin receptor 2C
5-HTT: Serotonin transporter
5-HTTLPR: Polymorphic region in the promoter region of 5-HTT
AUC: Area under the curve
CDT: Cold detection threshold
CPT: Cold pain threshold
DFNS: German Research Network on Neuropathic Pain
GAD: General anxiety disorder
HPT: Heat pain threshold
MDI: Major depression inventory
PCR: Polymerase chain reaction
PPT: Pressure pain threshold
QST: Quantitative sensory testing
SNP: Single nucleotide polymorphism
TG: Thermal grill
TRPV1: Transient receptor vanilloid 1
TSA: Thermal sensory analyzer
VAS: Visual analog scale
WDT: Warm detection threshold.
